# The most likely but largely ignored triggering factor for breast (or all) cancer invasion

**DOI:** 10.7150/jca.82291

**Published:** 2023-02-27

**Authors:** Yan-gao Man, Ciaran Mannion, Alexander Stojadinovic, George E Peoples, William CS Cho, Sidney W. Fu, Xiaohui Tan, Yi-Hsuan Hsiao, Aijun Liu, Andrzej Semczuk, Paul Zarogoulidis, Andrei B. Gapeev, Xiyun Deng, Xiaoning Peng, Boris A. Reva, Tatiana Omelchenko, Jialian Wang, Guohong Song, Tingtao Chen

**Affiliations:** 1 Department of Pathology, Hackensack Meridian School of Medicine, Nutley, NJ, USA; 2 LumaBridge, San Antonio, TX, USA; 3 Queen Elizabeth Hospital, Department of Clinical Oncology, Hong Kong, China; 4 Division of Genomic Medicine, Department of Medicine, and of Microbiology, Immunology & Tropical Medicine, George Washington University Medical Center, Washington DC, USA; 5 Department of Obstetrics and Gynecology, Changhua Christian Hospital, Changhua, Taiwan; 6 Department of Pathology, Chinese PLA General Hospital 7 th Medical Center, Beijing, China; 7 IIND Department of Gynecology, Lublin Medical University, Lublin, Poland; 8 Pulmonary-Oncology Department, "Theageneio" Cancer Hospital, Thessaloniki, Greece; 9 Laboratory of Biological Effects of Non-Ionizing Radiation, Institute of Cell Biophysics, Russian Academy of Sciences, Russian Federation; 10 Department of Pathology, Hunan Normal University School of Medicine, Changsha, Hunan, China; 11 Department of Genetics and Genomics Sciences, Institute for Genomics and Multiscale Biology, Icahn School of Medicine at Mount Sinai, New York, NY, USA; 12 Laboratory of Mammalian Cell Biology and Development, The Rockefeller University, New York, NY, USA; 13 Department of Sema4 Health Informatics, Stamford, CT, USA; 14 Department of Medical Oncology, Peking University Cancer Hospital and Institute, China; 15 Department of Gastrointestinal Surgery, The Second Affiliated Hospital of Nanchang University and National Engineering Research Center for Bioengineering Drugs and the Technologies, Institute of Translational Medicine, Nanchang University, Nanchang, China

**Keywords:** Breast myoepithelial cell layer, Tumor capsule, Tumor invasion, Cell interactions.

## Abstract

Breast cancer development and progression are believed to be a sequential process, from normal to hyperplastic, to *in situ*, and to invasive and metastatic stages. Given that over 90% of cancer deaths are caused by invasive and metastatic lesions, countless factors and multiple theories have been proposed as the triggering factor for the cascade of actions of cancer invasion. However, those factors and theories are largely based on the studies of cell lines or animal models. In addition, corresponding interventions based on these factors and theories have failed to reduce the incidence rate of invasive and metastatic lesions, suggesting that previous efforts may have failed to arm at the right target. Considering these facts and observations, we are proposing “A focal aberrant degeneration in the myoepithelial cell layer (MECL) as the most likely triggering factor for breast cancer invasion”. Our hypothesis is based on our recent studies of breast and multiple other cancers. Our commentary provides the rationale, morphologic, immunohistochemical, and molecular data to support our hypotheses. As all epithelium-derived cancers share a very similar architecture, our hypothesis is likely to be applicable to invasion of all cancer types. We believe that human tissue-derived data may provide a more realistic roadmap to guide the clinic practice.

## Editorial Commentary

All epithelium-derived cancers are architecturally similar with the epithelium (EP), capsule, and stroma 1,2. The EP is the origin of a majority of the human malignances 3,4. The breast capsule is made of the myoepithelial cell layer (MECL, a single cell layer embracing the EP) and basement membrane (BM, a thin layer of fibers and smooth muscle cells attached to the MECL) 5,6. The prostate and salivary tissues share similar capsule constitutes with the breast, whereas other cancers have only the BM constituting the capsule 7, 8. The stroma contains lymphocytic ducts, blood vessels, different immune cells, and EP cell metabolism-needed materials 9, 10.

**Figure 1:**
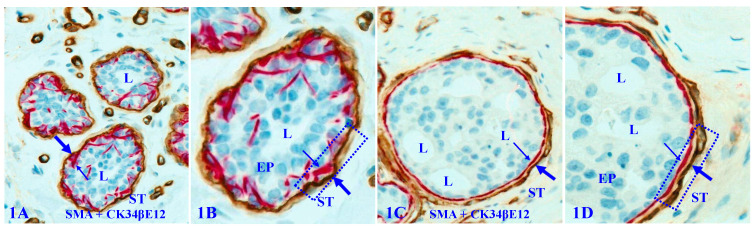
** Structural relationships of breast tissue components.** Formalin-fixed and paraffin-embedded human breast tissue sections are double immune-stained with a BM marker smooth muscle actin (SMA, brown) and ME cell marker CK34βE12 (red). B and D are a higher magnification of A and C. ST = stroma. EP = epithelium. L = Lumen. Thick arrows identify the BM. Thin arrows identify MECL. Squares identify capsules. Due to the conferment of the capsule, stem cell-derived proliferating cells are normally moving to the acinar or ductal lumen direction.

The capsule physically segregates the EP from the stroma and actively mediates the diffusion of EP growth- and metabolism-needed materials and nutrient from the stroma. In addition, as all types of EP cells belong to a self-renewal population and stem cells are normally located near the capsule, the capsule also functions as a physical confiner to force proliferating cells to migrate to the lumen and surface direction to replace aged or injured EP cells 11, 12. Figure 1 uses breast tissues as an example to elucidate the structural relationship of different EP tissue components.

**Figure 2:**
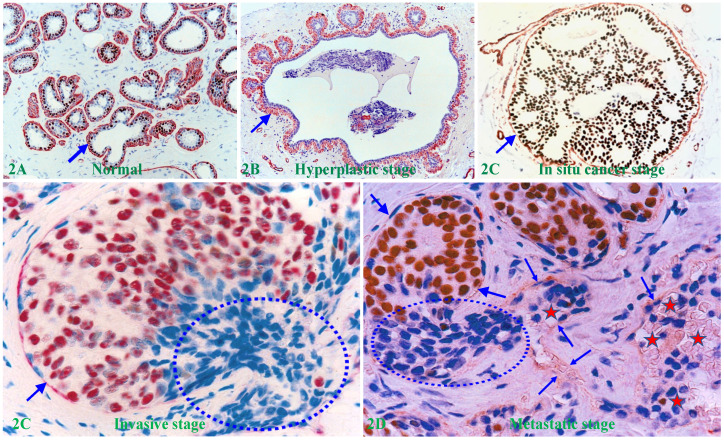
** The sequence of human breast cancer development and progression.** Formalin-fixed and paraffin-embedded human breast tissue sections were double immune-stained for ME cell marker smooth muscle actin (SMA, red) and estrogen receptor (ER, brown). Arrows identify the MECL. Circles identify EP cells overlying focally disrupted MECL and within the stroma (D). Red stars identify tumor and red blood cells within a blood vessel.

Human breast carcinogenesis is believed to follow the principle and steps proposed by “The clonal evolution theory", progressing sequentially from the normal to hyperplastic, to *in situ*, and finally to invasive and metastatic stages 13-15 (Figure 2).

With over 90% of cancer deaths resulting from invasion and metastasis-related illnesses 16-19, countless factors and theories have been proposed for triggering cancer progression:

1. Estrogen, progesterone, and their corresponding receptors, which have a dual role in regulating tumor cell proliferation and invasion 20-22.

2. Tumor suppressor genes, in which the reduction or lose can directly or indirectly lead to elevated migration and invasion of cancer cells 23-26.

3. Oncogenes, which cause aberrant expression of their oncoprotein products that facilitate tumor cell proliferation and migration 27-29.

4. Tumor dedifferentiation and dissociation, which mobilize the tumor cells out of the main tumor bulk and enables them to invade adjacent tissues by active locomotion 30-32.

5. Inflammatory signaling cascades, which are intimately involved in neoplastic processes fostering tumor cell proliferation, survival, invasion, and migration 33-35.

6. Capillary vessels derived from the periductal necklace of vessels, which breach the basement membrane, providing an escape hatch for cancer cell invasion 36-38.

7. Aberrant expression of P-Cadherin, which has been suggested as a stem cell marker associated with epithelial mesenchymal transition (EMT) and cancer invasion 39-41.

8. Tumor derived exosomes, which activate cancer-associated fibroblasts (CAFs) through miRNAs and Wnt pathway that in turn enhances invasion and metastasis 42-44.

9. Aberrant integrin expression, which supports oncogenic growth factor receptor (GFR) signaling and GFR-dependent cancer cell migration and invasion 45-47.

10. Mutational drivers, which promote chromosomal instability and genetic mutations that trigger cancer invasion 48-50.

11. Progressive changes in the structure and composition of tumor stroma, which is believed 14. to be a required transition to invasive breast cancer 51-53.

Unfortunately, these factors and theories are largely based on clinical testing results or results from *in vitro* studies on cancer cell lines or animal models. In addition, none of above factors or theories has elucidated the specific pathways for cancer cells to overcome the following physical and functional barriers for invading:

1. The ME or basal cells in breast, salivary and prostate gland, which embrace the entire EP system 54-56. How do the cancer cells physically cross over ME or basal cell layers?

2. The tumor capsule in other cancer types, which is composed of smooth muscle cells and dense fibers 57-59. How do the cancer cells physically breach these structures?

3. Intercellular junctions and adhesion molecules, which intercalate all EP cells into a single sheet 60-62. How do the cancer cells physically disassociate into individual cells?

4. The stromal and immune surveillance system, which harbors a variety of self-defensive cells 63-65. How do the invading cancer cells escape from this surveillance system?

5. The cancer stem cells, which are universally regarded as the direct precursor of invasive lesions 66-68. How do the stem cells physically enter the invasion cascade?

In addition to the factors and theories alluded above, the matrix metalloproteinase (MMP) family and the associated proteolytic enzyme theory were once universally considered to be the most likely factor and mechanism for triggering the invasion of all *in situ* cancer types 69-74. According to the proteolytic enzyme theory, aberrantly altered EP cells increasingly produce a wide variety of MMPs during their evolution, which reach the highest concentration at the *in situ* cancer stage. The elevated enzymes could selectively degrade the surrounding tumor capsule and intercellular adhesion molecules, resulting in disruptions or a total loss of the associated tumor capsule, which permits the cancer cells at the disrupted sites to freely migrate into their adjacent stroma or to invade lymphatic ducts or blood vessels 69-74 (Figure 2E).

The proteolytic theory was strongly supported by *in vitro* studies and animal models, which consistently showed that all matrix metalloproteinase family members could specifically degrade the tumor capsule and cause invasion, while the corresponding antagonists or neutralizing agents could partially or completely stop cancer invasion 75-81. Those findings inspired international efforts to develop such therapeutics for clinic trials in the late 1990's. However, thousands of the world-wide clinic trials with the corresponding antagonists or neutralizing agents of MMPs have failed to show any reduction of the cancer invasion rate 82-87. Those disappointing results of the world-wide clinic trials have led the world's top experts in the field to unanimously advocate the search for new directions and strategies to combat cancer invasion 83,88, 89.

Collectively, these facts have casted strong doubts on the validity of all proposed factors and theories on cancer invasion. Thus, we would like to propose that “A focal aberrant degeneration of the breast MECL (basal cells or capsule in other cancers) is the most likely but largely ignored triggering factor for cancer invasion” for following reasons:

## A. The MECL is closely associated with both carcinogenesis and cancer progression

### 1. The ME cell population possess a unique anti-cancer system

ME cells have a low rate of malignancies 90-93. Based on a 2013 article, “Myoepithelial carcinoma of the breast is extremely rare and only 33 cases have been reported in the English literature” 94. A 2020 article reported that “Breast mucoepidermoid carcinoma (MEC) is clinically rare, with an estimated incidence of 0.2-0.3% of all primary breast tumors” 95. It is likely that ME cells may possess a unique system that is resistant to carcinogenesis.

### 2. The MECL is the source of several types of tumor suppressors

**Figure 3:**
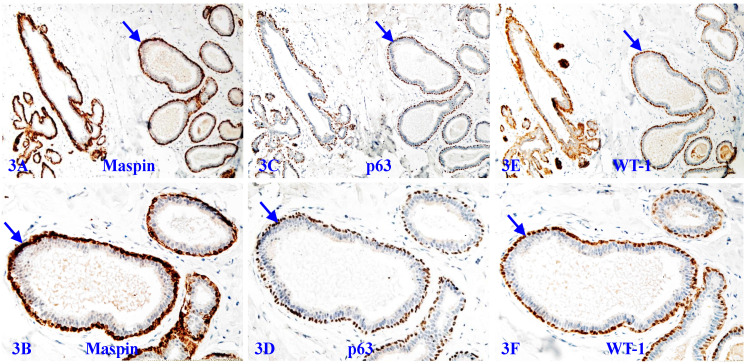
** Tumor suppressors in the MECL of normal and hyperplastic structures.** A set of three consecutive sections from a female patient with morphologically normal and hyperplastic appearing acinar and ductal structures were immune-stained for maspin, p63, and WT-1. Arrows identify the MECL. Please note that the MECL in all these acinar and ductal structures are non-disrupted and express high levels of all three tumor suppressors.

A number of studies have shown that the MECL produces a number of tumor suppressors, including p63, p73, Wilms' tumor 1 (WT-1), maspin, 14-3-3 sigma, and stefin A, which exert significant inhibition on the growth of tumor cells 96-101. The MECL also produces multiple proteinase- and angiogenic-inhibitors, which suppress cell migration 102-104. Figure 3 shows a set of 3-consecutive sections from a biopsy sample immune-stained for 3-different suppressors. It is apparent that they are co-expressed in the MECL of all normal and hyperplastic structures.

**Figure 4:**
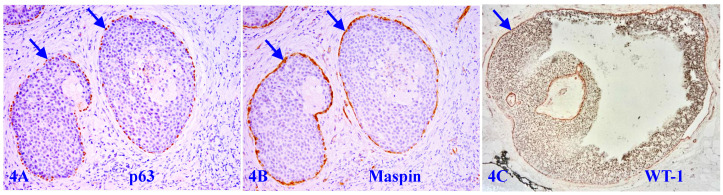
** Tumor suppressors in the MECL of ductal carcinoma *in situ* (DCIS).** Formalin-fixed and paraffin-embedded human breast tissue sections from two patients with ductal carcinoma *in situ* (DCIS) were immune-stained for p63, maspin, and WT-1. Please note that the MECL in all cases are non-disrupted and express high levels of all three tumor suppressors, even at the case with a large size of DCIS (4C).

The similar co-expression of different tumor suppressors is also seen in the MECL of a vast majority of DCIS cases, provided it is morphologically distinct and intact as shown in Figure 4.

### 3. Focal MECL disruptions confer epithelial cell invasive growth pattern

Breast EP cells are a self-renew population with stem cells normally located at the basal layer resting on the MECL 11,12. Due to the physical confinement imposed by the surrounding MECL and BM, stem cells normally undergo an orchestrated, progressive series of proliferation and differentiation steps at the basal layer, and then move unidirectionally upward towards the acinar or ductal luminal direction to replace aged or damaged cells. However, if the surrounding capsule is disrupted, the confinement force will be lost and proliferating cells will be more easily migrating to invade into the stroma. As the EP is normally devoid of lymphatic ducts and blood vessels, whereas the stroma is very rich in both, and thus, invading cells are prone to metastasize.

**Figure 5:**
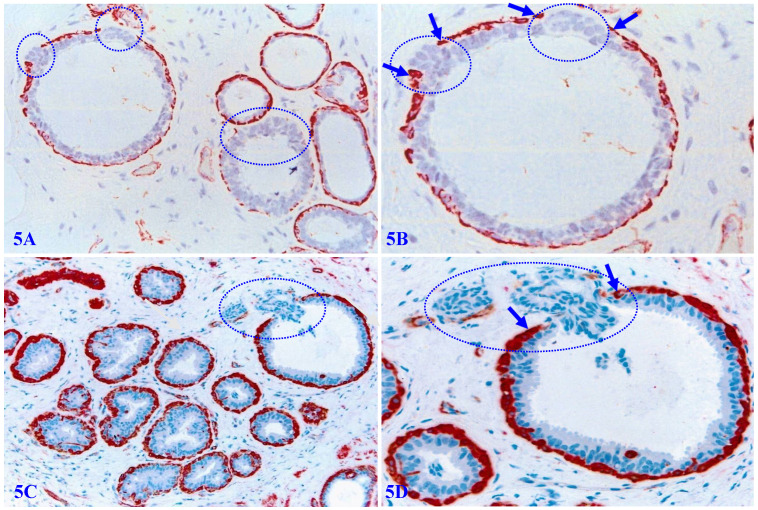
** Invasive growth pattern in normal and hyperplastic appearing breast ducts.** Formalin-fixed and paraffin-embedded human normal and hyperplastic appearing breast tissue sections were immune-stained for smooth muscle actin (SMA). Arrows identify residual ME cell layers. Circles identify cell clusters overlying focally disrupted MECL, which are arranged as tongue-like protrusions “invading” into the adjacent stroma.

Figure 5 shows a normal (A-B) and hyperplastic (C-D) appearing duct, which harbors three focal disruptions in the MECL (the absence of ME cells resulting in a gap larger than a combined size of at least 3 ME cells in at least 2 or more consecutive sections). EP cells overlying each of these focal MECL disruptions form a tongue-like protrusion “invading” towards the stroma.

### 4. Focal MECL disruptions selectively favor formation of ER (-) and Her-2 (+) cell clusters

**Figure 6:**
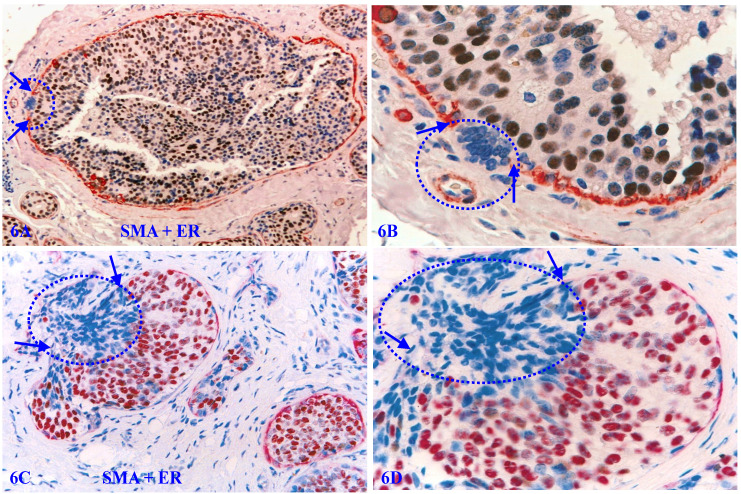
** ER-negative cell clusters overlying focally disrupted MECLs.** Formalin-fixed and paraffin-embedded human breast cancer tissue sections are double immune-stained for SMA (red) and ER (brown or pink). Arrows identify residual MECL. Circles identify ER-negative cell clusters overlying focal MECL disruptions. Please note that cells overlying focal MECL are ER-negative, whereas cells within the tumor core are ER-positive.

Our previous studies have consistently shown that the size of focal MECL disruptions along with the overlying tumor cell clusters in the pure cases of DCIS is very small and hard to find in H & E-stained sections 105-114. As shown in Figure 6A - 6B, a large DCIS harbors a small disruption with only about 10-overlying ER-negative cells. In contrast, the size of focal MECL disruptions and overlying tumor cells clusters are significantly larger in cases of invasive ductal carcinoma (IDC; Figure 6C-6D).

**Figure 7:**
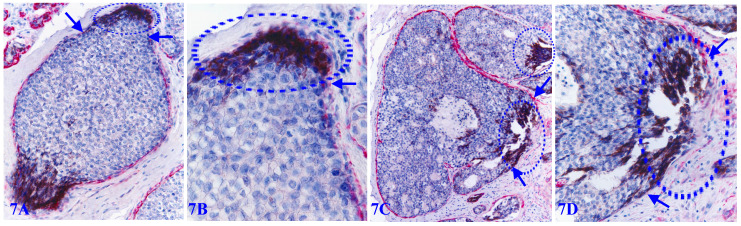
** Her-2 positive cell clusters overlying focally disrupted MECLs.** Formalin-fixed and paraffin-embedded human breast cancer tissue sections are double immune-stained for SMA (red) and Her-2 (black). Arrows identify residual MECLs. Circles identify Her-2-positive cell clusters overlying focal MECL disruptions. Please note that the EP cells within the tumor core surrounded by the residual MECL are largely devoid of Her-2 expression.

Focal MECL disruptions also facilitate the formation of Her-2 (+) cell clusters overlying focal MECL disruptions. As showed in Figure 7, EP cells surrounded by the residual MECLs are largely devoid of Her-2 expression, whereas all ductal EP cells localized at the focal MECL disruptions show high levels of cytoplasmic Her-2. Cells overlying focal MECL disruptions are arranged as tongue-like protrusions “invading” into the adjacent stroma.

### 5. Tumor cells overlying focal MECL disruption show a unique gene expression pattern

**Figure 8:**
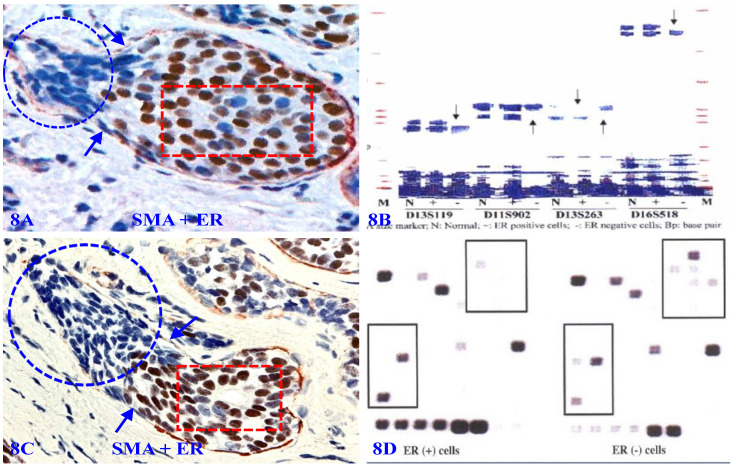
** Gene expression profiling of cells overlying focally disrupted MECLs.** Formalin-fixed and paraffin-embedded human breast tissue sections are double immune-stained for SMA (red) and ER (brown). Arrows identify the residual MECL near focal disruptions. Circles identify ER-negative cells overlying focal MECL disruptions. Squares identify ER positive cells still enclosed by residual MECL. Please note that ER-negative cells overlying focally disrupted MECL have a different LOH and gene expression profiles compared to their ER-positive counterparts within the tumor core.

Our previous studies have consistently shown that micro-dissected ER-negative cell clusters overlying focally disrupted MECL have a different rate and pattern of the loss of heterozygosity (LOH), and expression of cell proliferation and stem cell markers compared to their comparable counterparts within the same tumor core surrounded by the residual MECL 105-114 (Figure 8).

### 6. Focal MECL disruptions are associated with MMP- and ER-negative cell clusters

**Figure 9:**
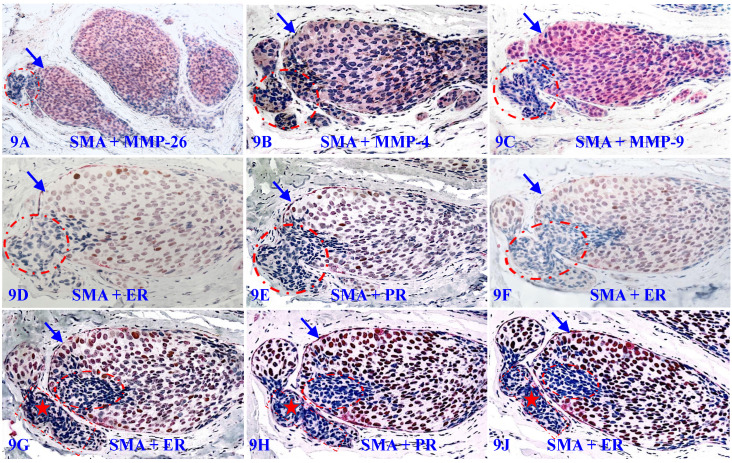
** Focal MECL disruptions are associated with MMPs- and ER-negative cell clusters.** A set of 9 consecutive sections of a DCIS were double immune-stained for SMA (brown or red) and MMP-26, MMP-4, MMP-9, ER, or PR. Circles identify focal MECL disruptions and overlying cells. Arrows identify residual MECLs. Red stars identify vascular-like structures. Please note that: (1) all cell clusters overlying focal MECL disruptions are MMPs- negative, but cells within the tumor core surrounded by the residual MECL are MMPs positive, and (2) all cell clusters overlying focally disrupted MECL are ER- and PR-negative, whereas their counterparts within the tumor core are ER- and PR-positive.

Our previous studies have extensively studied the impact of focal MECL disruptions on the expression of a wide variety of cancer invasion-related molecules in confirmed DCIS cases 105-114. Figure 9 shows the expression status of 9-consecutive sections of a DCIS case double immune-stained for SMA, MMP-26, MMP-4, MMP-9, ER, and PR, respectively.

It can be seen that a small DCIS harbors a relatively large focal disruption on its MECL (circles), in which overlying cells and their adjacent counterparts still surrounded by the residual MECL exhibit different immunohistochemical features. In the first 3-sections, cells surrounded by the residual MECL are positive for MMPs, while cells overlying focal MECL disruptions are devoid of all MMPs. In the remaining 6-sections, cells surrounded by the residual MECL show distinct ER- and PR-positivity, while cells overlying disrupted MECL are ER- and PR-negative.

In Figure 9C to 9F, cells overlying focal MECL disruptions are arranged as tongue-like protrusions in which all MMP-9-, ER, and PR-negative cells are physically joined together into a single unit that is morphologically and immunohistochemically indistinguishable. Please note that, in Figure 9G to 9J, ER-negative cells within the stroma appear to be within vascular-like structures, suggesting that cells overlying focally disrupted MECL are able to join with similar cells and vascular structures from adjacent tissues to form metastatic lesions.

### 7. Focal MECL disruptions are associated with elevated immune-cell infiltration

**Figure 10:**
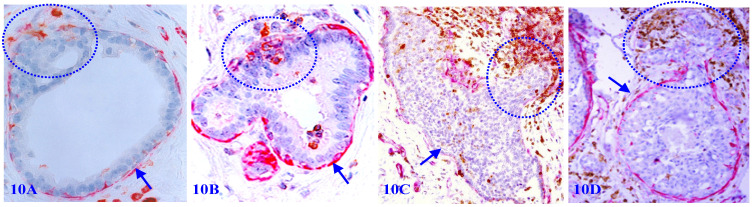
** Focal MECL disruptions are associated with elevated immune-cell infiltration.** Formalin-fixed and paraffin-embedded human breast tissue sections from 4 different cases were double immune-stained for SMA (red) and leukocyte common antigen (LCA; brown). Arrows identify the residual MECL. Circles identify infiltrating immune cells overlying focal MECL disruptions. Please note that the non-disrupted MECL is largely devoid of infiltrated immune cells.

Focal MECL disruptions among normal, hyperplastic, and pre-invasive cancers are almost always associated with elevated infiltration of immune cells, which are consistently located at or near disruptions. Non-disrupted MECLs are barely with immune-cell infiltration (Figure 10).

Collectively, these findings strongly suggest that the morphological integrity of the MECL has the crucial functions on the proliferation, growth, gene expression, migration of associated EP cells. These findings also strongly suggest that a focal MECL disruption has the potential to trigger a cascade of reactions of cancer invasion- and metastasis-related events.

## B. The MECL also suffers from a wide variety of pathologic alterations

On the other hand, in addition to the focal disruptions seen above, the MECL also suffers from a wide variety of pathologic or immunohistochemical alterations:

### 1. The loss of phenotypic marker SMS in morphologically distinct MECLs

**Figure 11:**
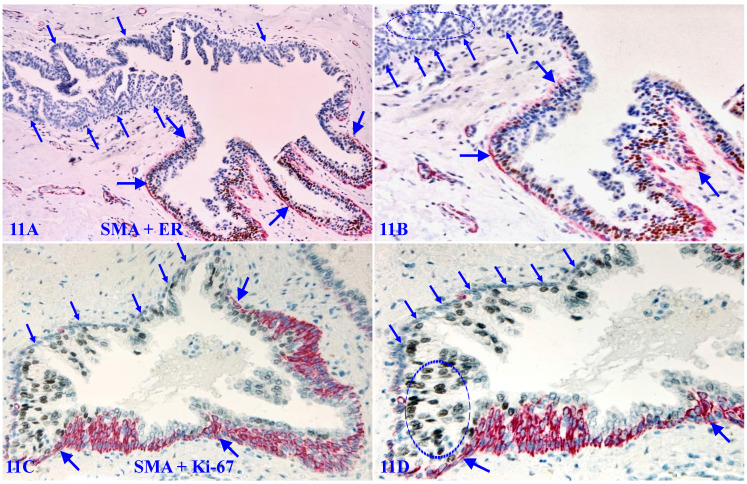
** Loss of phenotypic marker in morphologically distinct MECLs.** Formalin-fixed and paraffin-embedded human breast tissue sections from a DCIS case are double immune-stained for SMA (red) and ER or Ki-67 (black). Thick arrows identify the residual MECL. Thin arrows identify morphologically distinct MECLs that are devoid of the SMA expression. Circles identify ER-negative cells or Ki-67-positive EP cells. Please note that all SMA negative ME cells completely lack the expression of the proliferating cell specific marker Ki-67.

SMA is a normal and primary constitute of the MECL and BM, functioning as the core of the capsule of all EP-derived cancers, and double immunohistochemistry with SMA and cytokeratin has been routinely used to confirm early invasive breast and other cancers 115,116. However, a subset of cases harbor MECLs that are morphologically distinct, while are devoid of the SMA expression. As shown in Figure 11A - 11B, all EP cells surrounded by the SMA negative MECL are devoid of ER-expression, whereas their adjacent counterparts are largely ER-positive.

Figure 11C - 11D show an adjacent section from the same case double immune-stained for SMA and Ki-67, a cell proliferation specific marker. A vast majority of EP cells surrounded by SMA-negative MECL are strongly positive for Ki-67, while all SMA-negative ME cells are completely devoid of the Ki-67 expression, suggesting that these SMA-negative ME cells may have lost the power, or have had a unique system, of the self-cellular replenishment.

### 2. Elevated apoptosis in focally disrupted MECL

**Figure 12:**
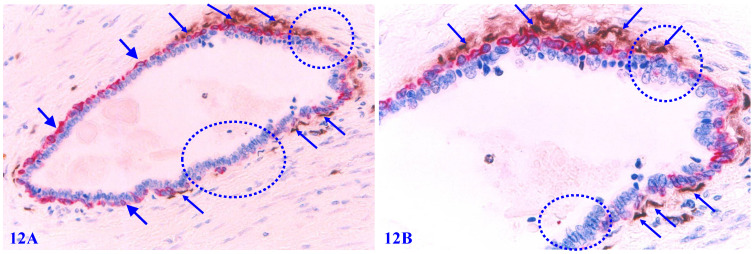
** Elevated apoptosis in focally disrupted MECLs.** Formalin-fixed and paraffin-embedded human breast tissue section was double immune-stained for SMA (red) and an apoptotic marker (brown). Thick arrows identify the residual MECL. Thin arrows identify apoptotic ME cells. Squares identify focal MECL disruptions. Please note that all apoptotic ME cells are located at or near focal MECL disruptions.

Among morphologically comparable EP structures, MECLs with focal disruptions also have a significantly higher rate of apoptosis. A vast majority of apoptotic ME cells are located at or near the focal MECL disruptions, and no distinct apoptotic EP cell is seen (Figure 12).

### 3. Elevated Tenascin expression in focally disrupted MECLs

**Figure 13:**
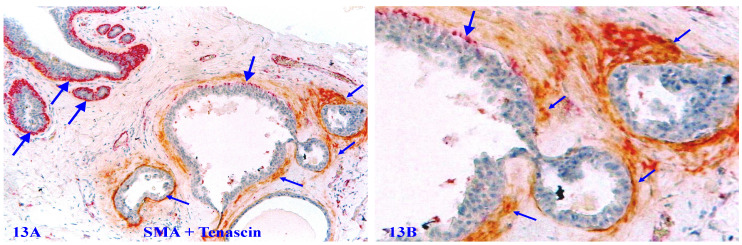
** Elevated Tenascin expression at focally disrupted MECLs.** Formalin-fixed and paraffin-embedded human breast tissue section was double immune-stained for SMA (red) and Tenascin (brown). Thick arrows identify non-disrupted or residual MECL. Thin arrows show the Tenascin positivity. Please note that a high level of Tenascin expression is seen at or near focally disrupted MECL, whereas the non-disrupted MECL is completely devoid of any distinct Tenascin expression.

Tenascin C is an extracellular matrix glycoprotein, which paves the paths and facilitates the migration and metastasis of breast cancer cells 117-119. Our previous studies have shown that Tenascin C is also highly expressed at distinct degenerating prostate basal cells, and EP cells in the vicinity of areas with elevated Tenascin C often lose the cohesion 120-125 (Figure 13).

### 4. Immunohistochemically altered MECLs in normal and hyperplastic tissues

In a vast majority of autopsy, biopsy, and surgically resected breast tissues, the MECL is physically continuous with a high level of tumor suppressor expression as those seen in figures 3 and 4 above. However, about 10-15% of cases harbor normal or hyperplastic tissue clusters that display several forms of morphological and immunohistochemical alterations in MECLs. These altered MECLs are generally distributed in the entire structures of a given small lobule.

**Figure 14:**
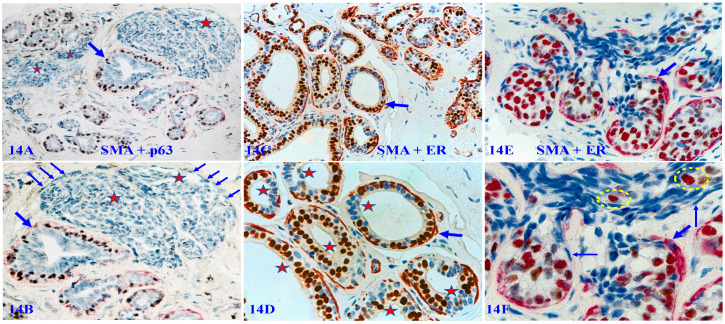
** Immunohistochemically altered MECLs in normal and hyperplastic tissues.** Formalin-fixed and paraffin-embedded human breast tissue sections from 3-cases were double immune-stained for SMA (red) and p63 (black), or ER (brown). Thick arrows identify residual MECLs. Thin arrows identify immunohistochemically altered ME cells. Stars identify solid tumor nests or ducts with focally disrupted MECL and ER-negative cells clusters. Circles identify ER- positive cells within a vascular-like structure. The MECLs in all structures of all 3-cases are immunohistochemically altered.

Figure 14A - 14B shows that all MECLs in the entire normal and hyperplastic structures are either focally disrupted or are devoid of the SMA expression in morphologically distinct ME cells of multiple solid tumor nests. Figure 14C - 14D shows that all MECLs in all normal and hyperplastic ducts within a lobule are focally disrupted with ER-negative cell clusters overlying focal MECL disruptions. Figure 14E - 14F shows that all MECLs in normal-appearing structures are focally disrupted with ER-negative and positive cells migrating into vascular-like structures.

### 5. Mixed normal appearing ME and EP cell clusters with cytoplasmic Her-2 expression

**Figure 15:**
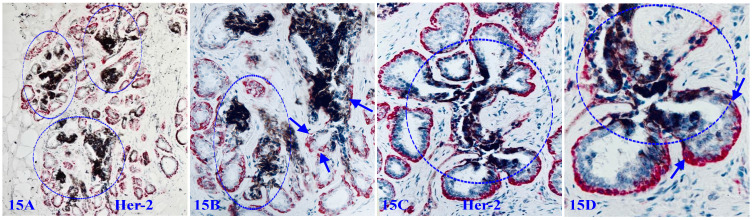
** Mixed normal appearing ME and EP cell clusters with cytoplasmic Her-2 expression.** Formalin-fixed and paraffin-embedded human breast tissue sections from two cases were double immune-stained for SMA (red) and Her-2 (black). Arrows identify normal appearing MECL. Circles identify focal MECL disruptions and overlying Her-2 positive cell clusters. Please note that these Her-2-positive cells are arranged as tongue-like protrusions invading into the stroma or vascular-appearing structures.

Some focal MECL disruptions are also associated with cytoplasmic Her-2 expression. As shown in Figure 15, these cytoplasmic Her-2 expressing cells are exclusively located at or near focal MECL disruptions, and often extending into tongue-like protrusions invading the stroma or vascular-like structures. These clusters and their derivatives are immunohistochemically and morphologically comparable to those seen in DCIS cases, suggesting that these cell clusters are very likely developed from the same progenitors and also have the same clinic significance.

Collectively, these findings suggest that the ME cell population belongs to a self-renewal population, which must constantly undergo cell proliferation and differentiation to replace aged or injured cells. Consequentially, any internal or external pathological insult on the ME cell population or its progenitors would undoubtedly have the potential to result in a wide variety of morphological and immunohistochemical alterations.

## C. Our detailed hypothesis of MECL-mediated cancer invasion

Based on above findings, we have hypothesized that breast cancer progression and invasion are triggered by the aberrant morphological and immunohistochemical alterations in the MECL via the following specific mechanisms and steps:

1. The predisposition of genetic defects in ME cell replenishment-related genes results in elevated focal degeneration in some ME cells (Figure 11-13);

2. The degradation products of ME cells attract the infiltration of immunoreactive cells into the affected sites to clean-up the degenerated ME cells and the BM (Figure 10);

3. The destruction and cleaning-up of degenerated ME cells and the BM results in a focal disruption in the MECL (Figure 10);

4. A focal MECL disruption results in a focal reduction or loss of tumor suppressors, which favors the proliferation of stem cells or an more aggressive cell clone (Figures 5-8);

5. A focal MECL disruption also results in the increase of oxygen, nutrients, and growth factors that promote stem cell-mediated EP cell proliferation;

6. The apoptosis and degeneration of ME cells promote the expression of Tenascin and Her-2, which facilitate the migration and invasion of proliferating EP cells (Figures 12-13);

7. The proliferation of a give stem cells generates a cluster of unmatured cells that lack the expression of cell age-dependent proteins, including ER, PR, and MMPs (Figure 9);

8. A focal MECL disruption lifts the confinement of the capsule, which selectively favors proliferating stem cells to migrate into the stroma (Figure 5-9);

9. A focal MECL disruption favors multipotent stem cells to form a mixed cell population with the capability to make its own lymphatic or vascular structures facilitating adjacent tumor cells to metastasize (Figure 14).

Compared to previously proposed theories of breast cancer invasion 20-53, our hypothesis is distinct in two main respects: (1) our hypothesis is entirely based on morphological, molecular, and immunohistochemical data from studies on human breast tissues with detailed clinic records and follow-up data, and (2) our hypothesis can reasonably elucidate the cellular and molecular mechanisms for cancer cells to overcome potential physical and functional barriers for invasion.

In comparison to the proteolytic enzyme theory of cancer invasion 69-88, our hypothesis is also noteworthy in that (1) our studies have consistently revealed that EP cells overlying focally disrupted MECL and their stromal derivatives are devoid of MMPs, while cells within the tumor core surrounded by the residual MECL have a high level of MMPs (Figure 9), and (2) “invasive” growth patterns are also seen in normal or hyperplastic appearing structures (Figures 5 and 14).

Our hypothesis also differs from the "clonal evolution and cancer heterogeneity theory", which proposes that "most neoplasms arise from a single cell of origin, and tumor progression results from acquired genetic variability within the original clone allowing sequential selection of more aggressive sublines" 13. Although the main concept of this theory has been universally accepted and supported by several lines of experimental and clinic data 126-135, it leaves four unanswered questions: (1) is carcinogenesis initiated by a normal or cancer stem cell? (2) is "sequential selection of more aggressive sublines" alone sufficient to accomplish carcinogenesis and progression? (3) can an aberrant microenvironment change the fate of "acquired genetic variability within the original clone"? (4) is the progression of different tumors triggered by the same or different factor(s)?

Our hypothesis is very likely to be applicable to the invasion cascade observed in all EP-derived malignancies. Our previous studies of human prostate, lung, gastric, colorectal, skin, salivary gland, and cervical tumor tissues have consistently seen that the capsules of these tissues (especially the prostate tumor) harbor morphologically and immunohistochemically similar focal disruptions, which are associated with EP cell alterations that are essentially identical as those seen in the breast 109, 110, 120-125.

A recent article compared our hypothesis with the enzyme theory, and has concluded that “…the FMCLD theory has some advantages over proteolytic theory because it focuses on the interaction of the different types of cells present in the tumor microenvironment 136. The localized death of myoepithelial cells causes the release of its inner contents like the proteolytic enzymes and growth factors. The resulting immunoreactions that accompany an external environmental insult or internal genetic alterations are triggering factors for further disruptions of the myoepithelial cell layer, BM degradation, and subsequent tumor progression and invasion”. Many other articles have also reported the advantages of our hypothesis and results 137-142.

## D. Specific applications of MECL in detection and interventions of cancer invasion

Based on above findings, it is apparent that the MECL not only decisively controls the proliferation rate and migration direction of adjacent EP cells, but also accurately reflects their functional status. More importantly, the MECL is intimately intermixed with the BM, forming the only physical barrier to inhibit the invasion of malignant EP cells into the stroma. As the morphologic, pathological, and immunohistochemical profiles of the ME cell population is far more easily recognizable and definable than its EP counterpart, the MECL appears to have the following specific clinic implications and applications:

1. **To use maspin and ER expression as independent risk factors for identifying more aggressive lesions and corresponding therapeutics.** Previous studies have shown that the expression of maspin is correlated with breast cancer invasion, brain metastases, and cancer recurrence 143-146. Previous studies have revealed that the ER-expression level is a reliable indicator for cancer therapeutic responses 147-150. Therefore, to assess the mRNA expression level of masoin and ER in the serum is likely to facilitate the detection of more aggressive lesions and the corresponding therapeutics.

2. **To use p63 as risk factor for a population-based screening to detect predisposition of cancer susceptibility or tumor suppressing genes.** Since p63 belongs to the p53 tumor suppressor family, and is normally expressed in the nucleus of the ME cells 151,152, an aberrant expression level or subcellular localization of p63 may signify the predisposition of cancer susceptibility or mutated suppressing genes. Previous studies have consistently revealed that the loss or cytoplasmic expression of p63 is associated with elevated stem cells, enhanced cell migration and metastasis, and increased mortality 153-155.

3. **To use ME cell-derived tumor suppressor-related signatures for early non-invasive detection of breast cancer.** Previous studies have consistently shown that several forms of tumor suppressor-related signatures are detectable in the blood sample and body fluid of patients with breast cancer 156-162. Thus, a statistical comparison of the expression levels of p63, maspin, and other tumor suppressors in the blood samples and body fluid may be used as a non-invasive clinic test or a population-based screening method for the detection of the specific individuals with or at a higher risk for early breast cancer.

4. **To search exfoliated ME cells or p63-related signatures in ductal lavage to identify women at increased genetic risk of breast cancer.** Previous studies have consistently shown that a variable number of exfoliated epithelial cells can be retrieved from ductal lavage for different assays for the identification of BRCA1/BRCA2 mutations 163-166. The search for exfoliated ME cells or p63-related signatures in ductal lavage may lead to the development of a new non-invasive cancer detection method.

5. **To use the MECL physical integrity (disrupted vs non-disrupted) as a clinic marker for the differentiation diagnosis.** As the disruption of the MECL is a prerequisite for breast cancer invasion and metastasis, the physical integrity of the MECL in patients with and without focal disruptions could effectively differentiate between non-invasive and invasive breast cancer.

6. **To use Tenascin expression in focally disrupted MECL as a routine clinic test of breast biopsy.** Previous studies have consistently demonstrated that aberrant Tenascin expression is exclusively seen at or near focally disrupted MECL and is also significantly correlated with breast cancer invasion and metastasis 117-119. Thus, the assessment of MECL-associated Tenascin expression may lead to the identification of the specific cases at increased risk for breast cancer progression.

7. **To use focal MECL disruptions as a localizer to identify cancer-stem cell clusters/ specific precursors of invasive cancer.** Our previous studies of multiple cancers have consistently shown that a focal disruptions of tumor capsules selectively facilitate clonal proliferation of overlying cancer stem cells to form distinct cell clusters. These newly formed clusters have a significantly higher level of cancer stem cell markers and invasion and metastasis-related gene expression than their morphologically comparable counters still enclosed by the non-disrupted tumor capsules 105-114 (Figure 8). It is very likely that these cell clusters may represent the direct precursors of invasive lesions. However, it is very difficult, if not impossible, to detect small MECL disruptions and associated ER negative cell clusters (Figure 6A -6B). Therefore, double immunohistochemistry with ER and SMA may be used as a reliable localizer to identify these potential stem cell clusters.

8. **To use MECL-associated immune cell infiltration to monitor the tumor progression and treatment responses.** Our previous studies have consistently shown that immune-cell infiltration is significantly associated with breast tumor capsule disruptions, which lead to the subsequent invasion and metastasis (Figure 10) 107, 109, 110, 120, 123. A number of recent studies have not only confirmed our previous reports and conclusions, but have also consistently shown that immune-cell infiltration is significantly correlated with the treatment responses in multiple cancer types 167-171. Therefore, a double immunohistochemistry with SMA and LCA to assess the physical integrity and extent of associated infiltrating immune cells of the MECL in the breast and the tumor capsule in other cancer types in biopsy samples may provide a novel clinic method to differentiate aggressive from indolent cancers, and also to monitor treatment responses of immuno- therapies.

9. **To use anti-inflammatory drug aspirin or statin to repair ME degeneration-related tumor capsule disruptions.** Previous studies have consistently demonstrated that aspirin or statin could significantly alter the chronic inflammation milieu of a variety of human cancers and prevent cancer progression 172-176. Thus, the administration of aspirin or statin to individuals with focally disrupted MECL associated with significant infiltration of immune cells (as shown in Figure 10) may potentially reduce the extent of infiltrating immune cells. It is likely that the reduction of the immune cell infiltration may facilitate the repairing of focally disrupted tumor capsules and consequently prevents the invasion of associated EP malignancies.

10. **To administer stem cell specific molecules, inducers, or stimulators to burst normal replenishment of MECL.** Previous studies have shown that a number of biomolecules, including CD24, CD44, CD133, Oct4, Gli1, ALDH1, Notch-1, Nectin-4, Neuregulin-1, Musashi-1, SSEA-3, DDX53, O-Acetyl-GD2, and DEAD-box helicase 27, are breast stem cell-related biomolecules, which are essential for the maintenance of the normal cellular replenishment or the regeneration processes after the internal or external insults 177-191. Thus, the administration of these molecules, or stem cell specific inducers and stimulators to patients with a high frequency of degenerations and focal disruptions (Figure 11, 14) may facilitate the restoration of the normal replenishment and functions of the MECLs. On the other hand, the administration of specific antagonists to those biomolecules may inhibit the aberrant EP cell proliferation and cancer stem cells-mediated invasion or metastasis.

11. **To use MECL lacking phenotypic markers or physically conjoined with vascular structures to identify novel cell proliferation pathways or cell cycle regulators.** As a subset of morphologically distinct and non-disrupted MECL is completely devoid of the expression tumor suppressors, phenotypic and proliferation specific markers (Figure 11) or is physically conjoined with vascular structures (Figure 14), it is likely that these ME cells are derived from or regulated by a previously unidentified mechanism or pathway 192-199. Thus, microdissection of these MECL for the gene expression profiling may lead to the identification of a novel cell proliferation pathway and cell cycle regulators.

12. **To use profiles of cells overlying focal MECL to explore therapeutic applications of "clonal evolution and cancer heterogeneity theory"^13^.** Although the main concept of the clonal evolution theory has been universally accepted and supported, it has failed to address four essential issues: (1) a normal or malignant nature of "single cell of origin" for carcinogenesis; (2) a partial or complete role of the EP in cancer development and progression; (3) a partial or complete role of microenvironment in cancer development and progression; (4) an all-shared or independent triggering factor for the invasion of all EP-derived cancers. The lack of definite answers for these fundamental issues makes it very difficult, if not impossible, to develop effective therapeutic strategies and agents for the early detection and intervention of the cancer invasion. Therefore, a systematic gene expression profiling and comparison of the profiles of the cells overlying focal MECL disruptions may lead novel findings that have the potential to address the unanswered issues and to develop more effective therapeutic strategies and agents.

In summary, the above findings strongly suggest that a focal disruption in the MECL of the breast or equivalent cell type and capsule in other EP-derived tissues is the most likely triggering factor for the cancer invasion. However, the impact of these EP-surrounding structures has been largely ignored since the previous efforts of the basic scientific researches on cancer invasion are primarily focused on cell lines or animal models. It is apparent that human tissue-derived basic research data may provide a more realistic and feasible roadmap to permit the observations of the direct interactions among different cell types, and thereby avoid potentially misleading pitfalls or shortcomings of *in vitro* and animal studies.
